# Tumor-specific memory CD8^+^ T cells are strictly resident in draining lymph nodes during tumorigenesis

**DOI:** 10.1038/s41423-023-00986-2

**Published:** 2023-03-01

**Authors:** Qiao Liu, Ling Ran, Zhengliang Yue, Xingxing Su, Lisha Wang, Shuqiong Wen, Shun Lei, Xiaofan Yang, Yan Zhang, Jianjun Hu, Jianfang Tang, Zhirong Li, Li Hu, Bo Zhu, Lifan Xu, Lilin Ye, Qizhao Huang

**Affiliations:** 1 Shanghai Public Health Clinical Center, Fudan University, Shanghai, China; 2 Institute of Cancer, Xinqiao Hospital, Third Military Medical University, Chongqing, China; 3 Institute of Immunology, Third Military Medical University, Chongqing, China; 4 Stomatological Hospital of Chongqing Medical University, Chongqing, China; 5 Provincial Key Laboratory of Immune Regulation and Immunotherapy, School of Laboratory Medicine and Biotechnology, Southern Medical University, Guangzhou, China; 6 College of Veterinary Medicine, Qingdao Agricultural University, Shandong, China; 7 Institute of Immunological Innovation and Translation, Chongqing Medical University, Chongqing, China

**Keywords:** Tumour immunology, Cellular immunity

The functional exhaustion of CD8^+^ T cells represents a fundamental hallmark of chronic viral infection and cancer and, in both scenarios, is driven by prolonged exposure to persistent cognate antigens in the context of an immunoinhibitory microenvironment. Exhausted CD8^+^ T cells upregulate the expression of a wide diversity of coinhibitory immunoreceptors (also referred to as immune checkpoint receptors), such as PD-1, Tim-3, LAG-3, and TIGIT. Concomitantly, exhausted CD8^+^ T cells lose their potential to differentiate into functional memory cells and are characterized by hierarchical loss of effector function, leading to compromised tumor control and viral eradication [1, 2].

Exhausted CD8^+^ T cells in the tumor microenvironment (TME) are highly heterogeneous, mainly consisting of subsets of TCF-1-expressing precursors of exhausted T (T_PEX_) cells and Tim-3-expressing terminally differentiated exhausted CD8^+^ T (T_EX_) cells [3, 4]. Immune checkpoint receptor blockade (ICB) therapies, such as those that block the PD-1/PD-L1 pathway, result in remarkable remission in a subset of cancer patients, with these effects generally attributed to the reversal of CD8^+^ T-cell exhaustion in the TME [5–7]. However, accumulating evidence highlights the potential role of systemic CD8^+^ T-cell responses in the control of tumor progression upon PD-1/PD-L1 ICB treatment, especially in tumor-draining lymph nodes (TdLNs) [8–14]. Recently, we reported on tumor-specific memory CD8^+^ T cells in TdLNs (TdLN-T_TSM_) of both mice tumor models and human hepatocellular carcinoma patients and showed that TdLN-T_TSM_ cells serve as primary responders to PD-1/PD-L1 ICB and exhibit superior tumor repression to that of TCF-1^+^ T_PEX_ cells [15].

TdLN-T_TSM_ cells were comparable to conventional memory T cells (T_MEM_) generated during acute viral infection in several aspects, including antigen-independent self-renewal and proliferation burst upon antigen reencounter. T_TSM_ cells express the lymphoid homing molecule L-selectin (CD62L) and C-C chemokine receptor 7 (CCR7). Moreover, we noticed that genes associated with T-cell extravasation and chemotaxis were less enriched in TdLN-derived P14 cells than in T_MEM_ P14 cells, indicating the potentially distinct circulating features between these two subsets [15]. T_MEM_ cells can patrol between lymphoid organs, blood and peripheral tissues, while during chronic viral infection, T_PEX_ cells are reported to largely reside in lymphoid tissues, with a very limited population in infected peripheral tissues [16]. Importantly, the migratory pattern of T_TSM_ cells derived from TdLNs during tumorigenesis has not yet been elucidated. Thus, herein, we sought to dissect the migratory pattern of T_TSM_ cells.

First, to precisely trace the immune response of TdLN-T_TSM_ cells during tumorigenesis, C57BL/6 mice (hereafter referred to as B6 mice) were first adoptively transferred with naive *Tcf7* (encoding TCF-1 protein)-GFP P14 cells (CD44^−^GFP^+^, GFP indicating TCF-1 expression) harboring transgenic TCRs specific to the H-2D^b^ Gp^33-41^ epitope from *Tcf7*-GFP knock-in reporter P14 mice, and then these B6 mice were subcutaneously inoculated with B16.F10 melanoma cells expressing the LCMV glycoprotein as a surrogate neoantigen (hereafter referred to as B16.Gp) as we previously reported [15]. Fourteen days later, TdLN-T_TSM_ P14 cells were sorted and retransferred into tumor-bearing recipients at Day 8 post B16.Gp inoculation (Supplementary Fig. S1a, b). Then, activated P14 cells derived from different tissues were analyzed. Eight days later, we noticed that CD44^+^P14 cells were more abundant in TdLNs than in other compartments of tumor-bearing mice (Fig. 1a, upper panel). Furthermore, we found that TCF1^+^TOX^−^ T_TSM_ P14 cells were more enriched in TdLNs than in other tissues, including those of the TME (Fig. 1a, lower panel). In addition, we noted that the majority of T_TSM_ cells in TdLNs were CD62L positive but negative for sphingosine-1-phosphate receptor-1 (S1PR1) expression; notably, S1PR1 mediates lymphocyte egress from LNs [17]. A fraction of T_TSM_ cells also expressed CD69 and CD103 (Fig. 1b), which are known markers for tissue resident memory T cells [18]. The unique expression pattern of these molecules may guarantee their retention within dLNs. Thus, we hypothesized that T_TSM_ cells are likely resident in TdLNs.

**Fig. 1:**
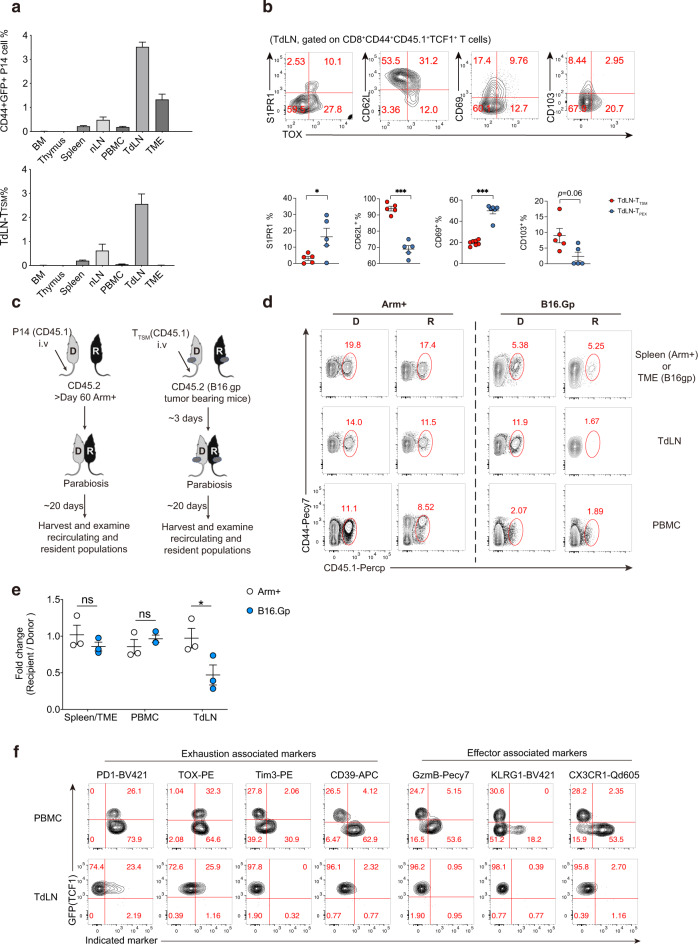
The in vivo distribution and migration pattern of tumor-specific memory CD8^+^ T cells during tumorigenesis. **a** Statistical summary of the proportions of CD45.1^+^CD44^+^ P14 cells (upper panel) and CD45.1^+^CD44^+^TCF1^+^TOX^−^ T_TSM_ cells (lower panel) in gated live lymphocytes from each compartment. BM bone marrow, nLNs nondraining lymph nodes, including axillary lymph nodes and submaxillary lymph nodes, TdLNs tumor-draining lymph nodes. *n* ≥ 4/group. **b** Flow cytometry analyses of TOX expression versus the indicated markers in CD45.1^+^CD44^+^TCF1^+^ donor P14 cells in TdLNs. **c** Experimental design of the parabiotic system. **d** Representative flow-cytometry plots of donor T_TSM_-derived CD44 + P14 cells in the TME, TdLNs, and PBMCs of the donor (D) and host/recipient (R) tumor-bearing parabionts (right panel). Donor-derived CD44+ P14 cells in the spleens, TdLNs, and PBMCs of donor- and recipient-infected parabionts are listed in the left panel. Numbers are frequencies. **e** The ratio of donor-derived CD44+ P14 cells from each indicated compartment in the recipient relative to the donor is summarized. **f** Flow-cytometry analyses of GFP (indicating TCF-1 expression) versus indicated exhaustion and effector cell-associated marker expression in CD45.1^+^CD44^+^ donor P14 cells from different compartments. *n* = 3/group. *p < 0.05 versus control, n.s. stands for not significant, paired two-tailed Student’s *t* test (**d**). Data are representative of 2 independent experiments (mean ± SEM)

To test this hypothesis, we next investigated the in vivo migratory properties of T_TSM_ cells by using a parabiotic system. T_TSM_ cells (CD45.1^+^CD45.2^−^CD44^+^PD-1^low^GFP^+^) were first sorted as previously reported [15] and transferred into tumor-bearing B6 recipients (CD45.1^−^CD45.2^+^). After resting for 3 days, the vasculature of B16.Gp tumor-bearing mice (adoptively transferred with T_TSM_ P14 cells on Day 6 post tumor implantation) were conjoined to those of tumor-matched mice (without P14 cell adoptive transfer) via parabiosis surgery. As a control, we performed the same surgery using LCMV-Arm^+^-acutely infected mice (P14 cells adoptively transferred) in which acute infection (>60 days) had cleared. Then, we tested whether donor P14 cells equilibrated between the parabionts 20 days later in the peripheral blood (PBMC), spleen (Arm^+^ infection), TME, and inguinal draining lymph node (Fig. 1c). As expected, virus-specific memory P14 cells established equilibrium between the two acutely infected conjoined parabionts in the spleen, peripheral blood, and inguinal lymph nodes, consistent with a previous report [16].

Notably, for tumor-bearing parabionts, donor (D)-derived P14 cells, including the T_TSM_ subset, were nearly undetectable in the TdLNs of recipient mice (R) (Fig. 1d, e). In contrast, donor-derived P14 cells reached equilibrium between parabionts in the tumor mass. Furthermore, we noticed that only a small fraction of donor-derived antigen-specific CD8^+^ T cells were recovered from peripheral blood, although the proportions seemed comparable between parabionts (Fig. 1d, e). This small population of antigen-specific CD8^+^ T cells in peripheral blood reminded us that during chronic LCMV infection (Cl13 infection), the frequency of tetramer-positive CD8^+^ T cells in the blood is very low compared to that in the spleen, and the majority of these circulating virus-specific CD8^+^ T cells in chronically infected mice were CD101^−^Tim-3^+^ CX3CR1^+^ transitory cells [16, 19–22]. To further characterize the progeny cells in the blood, we compared the phenotype of PBMC- and tumor-derived CD44^+^P14 cells from the host parabiont with those from the TdLNs of donor mice. Consistent with published data [15], antigen-specific CD8^+^ T cells in TdLNs consisted of TCF1^+^TOX^−^ T_TSM_ cells and a small proportion of TCF1^+^TOX^+^ T_PEX_ cells. In contrast, in PBMCs, a substantial proportion (~70%) of T_TSM_-derived P14 cells differentiated into TCF1-negative cells and upregulated the expression of exhaustion-associated markers, including PD-1, TOX, and CD39 (Fig. 1f). Furthermore, T_TSM_-derived P14 cells in PBMCs had highly increased expression levels of the chemokine receptor CX3CR1 and effector molecules KLRG1 and granzyme B, while these markers were barely expressed by TCF-1-expressing T_TSM_ cells in the draining lymph nodes (Fig. 1f), suggesting that antigen-specific CD8^+^ T cells in the peripheral blood were heterogeneous and in a transitory differentiation stage between progenitor and terminal exhausted CD8^+^ T cells. Additionally, T_TSM_-derived P14 cells primarily differentiated into TCF1-negative cells in the TME, with high levels of PD-1, TOX, Tim3, and CD39 expression (Supplementary Fig. S1c).

Collectively, our study delineated the unique tissue distribution and different migratory patterns of T_TSM_ and T_MEM_ cells. TdLN-T_TSM_ cells were predominantly found in TdLNs and seemed to be resident at these sites. Importantly, this study indicates that TdLNs might provide a unique niche in facilitating the differentiation and residency of TdLN-T_TSM_ cells. However, more efforts are needed to further explore this possibility and examine how such lymphoid niches are generated and operated during tumorigenesis.

## Supplementary information

Materials and Methods

Supplementary Table

Supplementary Figure

Supplementary Figure Legend
