# Author Correction: Tumor-specific memory CD8^+^ T cells are strictly resident in draining lymph nodes during tumorigenesis

**DOI:** 10.1038/s41423-023-01025-w

**Published:** 2023-05-25

**Authors:** Qiao Liu, Ling Ran, Zhengliang Yue, Xingxing Su, Lisha Wang, Shuqiong Wen, Shun Lei, Xiaofan Yang, Yan Zhang, Jianjun Hu, Jianfang Tang, Zhirong Li, Li Hu, Bo Zhu, Lifan Xu, Lilin Ye, Qizhao Huang

**Affiliations:** 1 Shanghai Public Health Clinical Center, Fudan University, Shanghai, China; 2 Institute of Cancer, Xinqiao Hospital, Third Military Medical University, Chongqing, China; 3 Institute of Immunology, Third Military Medical University, Chongqing, China; 4 Stomatological Hospital of Chongqing Medical University, Chongqing, China; 5 Provincial Key Laboratory of Immune Regulation and Immunotherapy, School of Laboratory Medicine and Biotechnology, Southern Medical University, Guangzhou, China; 6 College of Veterinary Medicine, Qingdao Agricultural University, Shandong, China; 7 Institute of Immunological Innovation and Translation, Chongqing Medical University, Chongqing, China

**Keywords:** Tumour immunology, Cellular immunity

Correction to: *Cellular & Molecular Immunology* 10.1038/s41423-023-00986-2, published online 01 March 2023

In this article, the affiliation details were incorrectly given as ‘^1^Institute of Immunology, Third Military Medical University, Chongqing, China. ^2^Institute of Cancer, Xinqiao Hospital, Third Military Medical University, Chongqing, China. ^3^Shanghai Public Health Clinical Center, Fudan University, Shanghai, China’ but should have been ‘^1^Shanghai Public Health Clinical Center, Fudan University, Shanghai, China. ^2^Institute of Cancer, Xinqiao Hospital, Third Military Medical University, Chongqing, China. ^3^Institute of Immunology, Third Military Medical University, Chongqing, China.’ While the author should be accordingly changed from Qiao Liu^1,2,3,8^, Ling Ran^1,8^, Zhengliang Yue^1,8^, Xingxing Su^1^, Lisha Wang^1^, Shuqiong Wen^4^, Shun Lei^1^, Xiaofan Yang^5^, Yan Zhang^6^, Jianjun Hu^1^, Jianfang Tang^1^, Zhirong Li^1^, Li Hu^1^, Bo Zhu^2^, Lifan Xu^1,*^, Lilin Ye^1,**^ and Qizhao Huang^7,***^ to “Qiao Liu^1,2,3,8^, Ling Ran^3,8^, Zhengliang Yue^3,8^, Xingxing Su^3^, Lisha Wang^3^, Shuqiong Wen^4^, Shun Lei^3^, Xiaofan Yang^5^, Yan Zhang^6^, Jianjun Hu^3^, Jianfang Tang^3^, Zhirong Li^3^, Li Hu^3^, Bo Zhu^2^, Lifan Xu^3,*^, Lilin Ye^3,**^ and Qizhao Huang^7,***^”

The original article has been corrected.

