# Supplementary material for: Tumor-specific memory CD8+ T cells are strictly resident in draining lymph nodes during tumorigenesis
Source: Cell Mol Immunol. 2023 Mar 1;20(4):423–6. doi: 10.1038/s41423-023-00986-2 (PMC10066293; doi:10.1038/s41423-023-00986-2)
Supplement: Supplementary file 1 — Materials and Methods [file 41423_2023_986_MOESM1_ESM.docx]

**Materials and Methods**

**Mice, virus infection, and tumor challenge.** CD45.1 and CD45.2 mice were purchased from Jackson Laboratory. P14 transgenic mice (specifically recognizing LCMV glycoprotein-derived peptide GP33-41 presented by H-2D^b^) were kindly provided by Rafi Ahmed (Emory University). All mouse strains were on a C57BL/6J background and were housed and bred under specific pathogen-free conditions. Mice have been infected with LCMV Armstrong strain [2 × 10^5^ PFU, intraperitoneally (i.p.)] for acute infection at 6-10 weeks of age. For the tumor model, mice were subcutaneously (s.c.) inoculated with 0.5 × 10^6^ B16.Gp (B16F10 cells expressing the lymphocytic choriomeningitis virus (LCMV) glycoprotein) tumor cells. All mice experiments were performed following the guidelines of the Institutional Animal Care and Use Committees of the Army Medical University.

**Adoptive T cell transfer.** For memory CD8^+^T cell parabiosis surgery, 5 ×10^4^ CD45.1+ naive splenic P14 cells were adoptively transferred into recipient mice with different congenic markers (CD45.2+) one day before LCMV infection. For sorting T_TSM_ cells, 5 × 10^5^ naïve CD45.1^+^ splenic *Tcf7*-GFP P14 cells were first adoptively transferred into naïve B6 mice, then inoculated with B16.Gp tumor cells one day later. 14 days later, CD45.1^+^ T_TSM_ were sorted as Live CD8^+^CD44^+^ CD45.1^+^GFP^+^PD-1^low^ and retransferred into tumor-bearing mice(3 × 10^5^/mice).

**Parabiosis Surgery.** Parabiosis surgery was performed as previously described(20) with minor modifications. Mice were anesthetized intraperitoneally with 4 mg of avertin 3 days after transfer. The anesthetized mice were placed onto the sterilized surgery hood and the drying preventive ointment was applied to the mouse's eyes. The left(CD45.1^+^ P14 transferred donor mouse) or right flank of the recipient mouse was shaved with an electric shaver and a depilatory cream to remove the remaining hair. Following disinfection, a mirrored incision was made on the lateral aspect of each mouse (from the olecranon to the knee joint), and a 4-0 Vicryl suture was used to sew the skin to conjoin the mice with interrupted suture. Additional sutures were placed through the olecranon and knee joints to secure the legs. Parabionts were then allowed to rest for 20 days to allow vascular connection and circulation equilibration before experiments.

**Preparation of single-cell suspensions from mouse samples.** Splenocytes, lymph node cells, PBMC, and TME cell (tumor microenvironment infiltrating lymphocytes) isolation were acquired as previously described(21). In brief, tumors were excised and manually dissociated. Hematopoietic cells and lung lymphocytes were further purified using a discontinuous Percoll gradient (GE Healthcare). Cells at the interface were harvested and washed twice before further use. Suspensions of spleen, thymus and lymph node cells were obtained by mashing the spleen, thymus, or lymph nodes through a 70mm nylon cell strainer (BD Falcon), axillary lymph nodes and submaxillary lymph nodes were used as non-draining lymph nodes.

**Flow Cytometry.** Flow cytometric analysis was performed on a FACSFortesa instrument (BD Biosciences) as previously described(22). All the antibodies used for flow cytometry were listed in supplementary table 1. Surface staining was performed in PBS containing 2% bovine serum albumin or FBS (w/v). Cells were stained with surface antibody cocktails for 30 min on ice. For the detection of transcription factors such as TCF-1, and TOX, surface-stained cells were permeabilized, fixed, and stained by using the Foxp3/Transcription Factor Staining Buffer Set (eBioscience, 00-5523) according to the manufacturer’s instructions.

**Statistical Analysis.** Statistical analysis was conducted with Prism 7 software (GraphPad software). Statistical differences were assessed using a two-tailed unpaired Student’s t-test. p values < 0.05 were considered significant (*: p < 0.05; **: p < 0.01; ***: p < 0.001); p values > 0.05: non-significant (ns).
