# Supplementary material for: Tumor-specific memory CD8+ T cells are strictly resident in draining lymph nodes during tumorigenesis
Source: Cell Mol Immunol. 2023 Mar 1;20(4):423–6. doi: 10.1038/s41423-023-00986-2 (PMC10066293; doi:10.1038/s41423-023-00986-2)
Supplement: Supplementary file 2 — Supplementary Table [file 41423_2023_986_MOESM2_ESM.docx]

## Supplementary Table

## Antibodies and reagents used

| **Antibody target/Reagent** | **Clone** | **Dilution** | **Provider** |
| --- | --- | --- | --- |
| CD8 Brilliant Violet 510™ | 53-6.7 | 1:200 | Biolegend |
| CD44 PE/Cyanine7 | IM7 | 1:200 | eBioscience |
| CD45.1 PerCP | A20 | 1:200 | Biolegend |
| LIVE/DEAD Fixable Near- IR Dead Cell Stain Kit | / | 1:200 | Life Technologies |
| PD-1 BV421 | 29F.1A12 | 1:150 | Biolegend |
| TOX PE | TXRX10 | 1:100 | eBioscience |
| Tim3 PE | B8.2C12 | 1:100 | Biolegend |
| CD39 APC | Duha59 | 1:100 | eBioscience |
| GzmB Pe-cy7 | QA16A02 | 1:100 | eBioscience |
| KLRG1 BV421 | 2F1/KLRG1 | 1:100 | eBioscience |
| CX3CR1 BV605 | SA011F11 | 1:100 | BD Biosciences |
| S1PR1 APC | SW4GYPP | 1:100 | Invitrogen |
| CD62L BV605 | MEL-14 | 1:100 | Biolegend |
| CD69 Pe-cy7 | H1.2F3 | 1:100 | Biolegend |
| CD103 PE | QA17A24 | 1:100 | Biolegend |
