# Supplementary figures and images for: Tumor-specific memory CD8+ T cells are strictly resident in draining lymph nodes during tumorigenesis
Source: Cell Mol Immunol. 2023 Mar 1;20(4):423–6. doi: 10.1038/s41423-023-00986-2 (PMC10066293; doi:10.1038/s41423-023-00986-2)

**a**

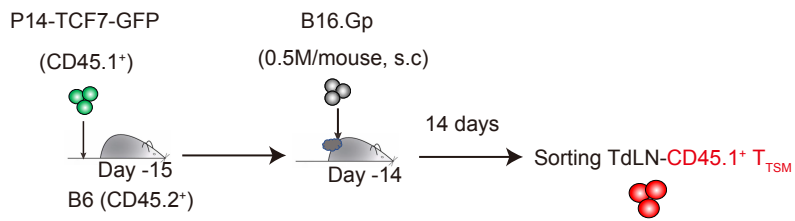

**b**

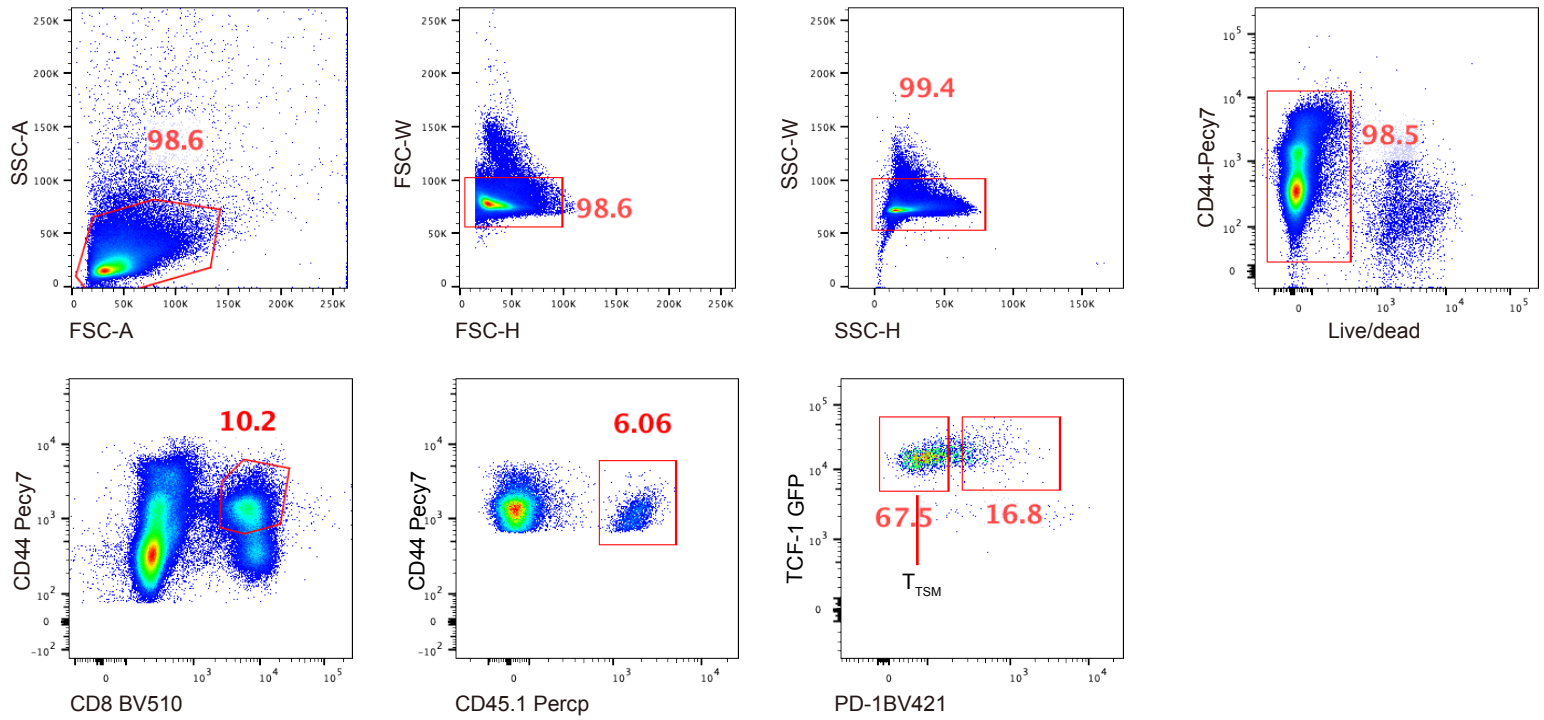

**c**

(Gated on CD8<sup>+</sup>CD44<sup>+</sup>CD45.1<sup>+</sup> T cells)

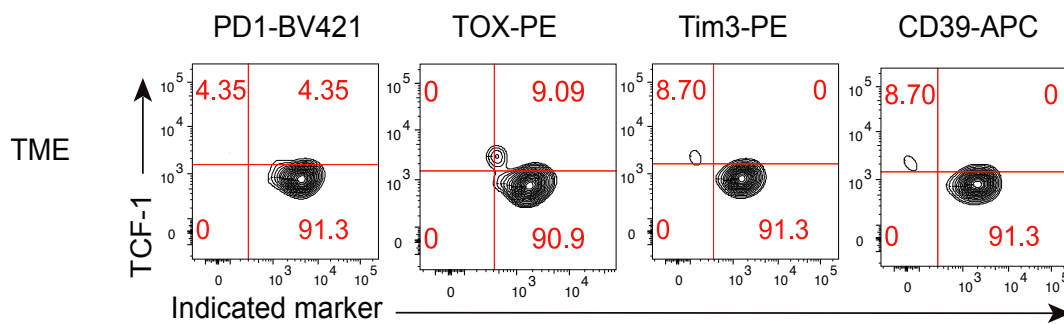

Supplement: Supplementary file 3 — Supplementary Figure [file 41423_2023_986_MOESM3_ESM.pdf]
