# Supplementary material for: Tumor-specific memory CD8+ T cells are strictly resident in draining lymph nodes during tumorigenesis
Source: Cell Mol Immunol. 2023 Mar 1;20(4):423–6. doi: 10.1038/s41423-023-00986-2 (PMC10066293; doi:10.1038/s41423-023-00986-2)
Supplement: Supplementary file 4 — Supplementary Figure Legend [file 41423_2023_986_MOESM4_ESM.docx]

**Fig. S1** **Experimental design and strategy** **for sorting TdLN-T_TSM_ cells_._**

**a**. C57BL/6 mice were first adoptively transferred with naive *Tcf7*-GFP P14 cells, and then these B6 mice were subcutaneously challenged with B16.F10 melanoma cells expressing the LCMV glycoprotein epitope; 14 days later, mice were sacrificed, and T_TSM_ cells from draining lymph nodes were sorted. **b**. Gating strategy for sorting TdLN-T_TSM_ cells. c. Flow-cytometry analyses of TCF1 versus indicated exhaustion-associated marker expression in CD45.1^+^CD44^+^ donor P14 cells within the TME of recipient mice.
